# Effect of the different 13-valent pneumococcal conjugate vaccination uptakes on the invasive pneumococcal disease in children: Analysis of a hospital-based and population-based surveillance study in Madrid, Spain, 2007-2015

**DOI:** 10.1371/journal.pone.0172222

**Published:** 2017-02-16

**Authors:** Juan Picazo, Jesús Ruiz-Contreras, Juan Casado-Flores, Sagrario Negreira, Fernando Baquero, Teresa Hernández-Sampelayo, Enrique Otheo, Cristina Méndez, On behalf of Heracles Study Group

**Affiliations:** 1 Medicine Department, School of Medicine, Universidad Complutense, Madrid, Spain; 2 Pediatric Department, Hospital Universitario 12 de Octubre, Madrid, Spain; 3 Pediatric Department, School of Medicine, Universidad Complutense, Madrid, Spain; 4 Pediatric ICU, Hospital Universitario Infantil Niño Jesús, Madrid, Spain; 5 Pediatric Department, School of Medicine, Universidad Autónoma, Madrid, Spain; 6 Pediatric Department, Hospital Universitario La Paz, Madrid, Spain; 7 Pediatric Department, Hospital General Universitario Gregorio Marañón, Madrid, Spain and CIBER of Respiratory Diseases, CIBERES, Madrid, Spain; 8 Pediatric Department, Hospital Universitario Ramón y Cajal, Madrid, Spain; 9 Medical Department, Pfizer SLU, Madrid, Spain; Universidade de Lisboa Faculdade de Medicina, PORTUGAL

## Abstract

In the Community of Madrid, the 13-valent pneumococcal conjugate vaccine (PCV13) replaced the 7-valent (PCV7) in the fully government-funded Regional Immunization Program (RIP) in May, 2010, but was later excluded in May, 2012, and included again in January, 2015. These unique changes allowed us to assess the impact of the different pneumococcal vaccination policies on PCV13 uptake in infants and on the incidence rate (IR) of invasive pneumococcal disease (IPD) in children <15 years old. In this prospective, active, surveillance study, we estimated PCV13 uptakes, IR and incidence rate ratios (IRR) for total IPD and for IPD caused by PCV13- and non-PCV13 serotypes in children <15 years, stratified by age, in four periods with different vaccination policies: fully government-funded PCV7 vaccination, fully government-funded PCV13, mixed public/private funding and only private funding. Vaccine uptakes reached 95% in periods with public-funded pneumococcal vaccination, but fell to 67% in the private funding period. Overall, IR of IPD decreased by 68% (p<0.001) in 2014–15, due to 93% reduction in the IR of PCV13-type IPD (p<0.001) without significant changes in non-PCV13-type IPD. A fully government-funded PCV13 vaccination program lead to high vaccine uptake and dramatic reductions in both overall and PCV13-type IPD IR. When this program was switched to private PCV13 vaccination, there was a fall in vaccine coverage and stagnation in the decline of PCV13-type IPD with data suggesting a weakening of herd immunity.

## Introduction

The introduction of pneumococcal conjugate vaccines (PCVs) in childhood immunization programs has markedly decreased the incidence of invasive pneumococcal disease (IPD) [1–4]. Data from USA in the pre- (1998) and post-vaccine (2014) era showed a drastic reduction of incidence rates in all age groups, with reductions of IPD incidence/100,000 inhabitants in children <12 months (from 165.3 to 15.9), 12–24 months (from 202.5 to 10.3), 2–4 years (from 36.9 to 6.3) and 5–17 years old (from 4.0 to 1.4) [5,6]. Reductions in IPD were accompanied by reductions in mortality rates and in non-susceptibility to penicillin (from 24.4% to 5.2%) and cefotaxime (from 14.1% to 3.4%) [5,6]. Marked reductions have also been reported in other countries as South Africa, where incidence of IPD after PCV introduction was 69% reduced, from 54.8 cases/100,000 inhabitants in the baseline period to 17.0 cases/100,000 inhabitants in 2012 [7].

Adherence to recommended vaccine schedules and high uptake are the most important factors for both individual protection and maintaining wider herd immunity in the community [8]. Reductions in IPD were associated with profound changes in pneumococcal nasopharyngeal colonization in the post-vaccination era since vaccine serotypes have been greatly reduced or nearly eliminated [9–11]. High vaccination coverages could limit circulation within the community of pneumococcal serotypes with greater capacity to cause invasive disease (vaccine types) being replaced by serotypes with lower invasive capacity, as has been hypothesized in relation to otitis media [12].

In October 2006, the seven-valent PCV (PCV7) was introduced into the Regional immunization program (RIP)—a public funding immunization program in the Autonomous Region of Madrid (≈6 million inhabitants). In June 2010, the 13-valent conjugate vaccine (PCV13) replaced PCV7 with a schedule of doses at 2, 4, and 12 months of age but it was later excluded from the RIP in 2012, because of changes in vaccination policies for children born after the 1^st^ of May of 2012. Finally, PCV13 was reintroduced in March 2015 for infants born after the 1^st^ of January of 2015. From May, 2012 to May, 2015, the vaccine was available at private markets for purchase by parents.

In the Region of Madrid, IPD in children <15 years of age has been the object of active surveillance since 2007, coinciding with the introduction of PCV7 in RIP. In this study, we aimed to assess the impact of different immunization policies on both vaccine coverage and IPD incidence.

## Materials and methods

### Study design

A prospective, active, population-based and clinical surveillance study on laboratory-confirmed IPD was carried out in all hospitals with pediatric departments located in the Autonomous Region of Madrid, Spain, from May, 2007 onwards [13–17]. A network of pediatricians, pediatric infectious disease specialists and microbiologists from all public and private hospitals in Madrid (27 hospitals) was created to carry out the active surveillance. Following the Spanish legislation, the present study was approved by the Research Ethics Committee of Hospital Clínico Universitario San Carlos (Madrid) and the approval was communicated to the Research Ethics Committee of all participating centers offering them the possibility for further review/clarifications. Written informed consent was obtained from parents/guardians before inclusion.

Data were analyzed annually: 1^st^ period (May 07 –April 08), 2^nd^ period (May 08 –April 09), 3^rd^ period (May 09 –April 10), 4^th^ period (May 10 –April 11), 5^th^ period (May 11 –April 12), 6^th^ period (May 12 –April 13), 7^th^ period (May 13 –April 14), and 8^th^ period (May 14 –April 15). Data from periods 1 to 5 were published [13–17]; however, in order to assess the impact of the different immunization policies on IPD incidence, data from all the study periods were re-distributed and re-analyzed. Four periods were considered based on the different pneumococcal vaccination policies as follows: PCV7 period (1^st^, 2^nd^ and 3^rd^ periods, with free PCV7 vaccination in RIP), PCV13 period (4^th^ and 5^th^ periods, with PCV13 included in the RIP), mixed public/private period (6^th^ period in which free PCV13 vaccination was withdrawn from RIP for children born on or after the 1^st^ of May of 2012, with free PCV13 universal vaccination for children born before this date to complete a 2+1 vaccination schedule), and private period (7^th^ and 8^th^ periods, with PCV13 vaccination paid for exclusively by children’s families).

Clinical forms of IPD (primary bacteremia, meningitis, empyema, bacteremic pneumonia and others) and demographic data (age, gender, pneumococcal vaccination status) were prospectively collected from children <15 years of age with laboratory confirmed IPD. IPD was defined as the presence of *Streptococcus pneumoniae* in normally sterile fluids, demonstrated by culture and/or polymerase chain reaction (PCR). Samples were sent to the clinical microbiology laboratory at each center for microbiological culture and/or PCR detection. All pneumococcal isolates were sent to a single reference laboratory (*Microbiology Department of the University Clinic Hospital in Madrid*) for serotyping by Quellung reaction. When pleural or cerebrospinal fluids from children with meningitis or pleural empyema did not yield positive cultures, samples of these fluids were also sent to the reference laboratory for PCR detection of pneumolysin (*ply*) and autolysin (*lyt*) genes [18,19]. Pneumococci confirmed by PCR were serotyped by real-time PCR assay using the LightCycler SYBR green format followed by melting-curve analysis as previously described [20], detecting serotypes 1, 3, 4, 5, 6, 7F, 14, 19A and 19F.

PCV13 serotypes were considered serotypes included in PCV13 and serotype 6C based on previously reported cross-protection data [21]. All other serotypes were considered non-PCV13 serotypes.

### Statistics

Annual population data on children <15 years old and estimated person-years data for different age groups (<24 months, 24-≤59 months, and >59 months) were obtained from the Spanish *Instituto Nacional de Estadística* (Spanish Statistical Office) [22].

Details of the number of PCV7 and PCV13 doses delivered per year in Madrid were obtained from IMS Health (Intercontinental Marketing Services, Madrid, Spain) as total vaccine sales per year. Yearly estimated vaccine coverage (assuming a 2+1 dose schedule) was calculated by dividing total doses by number of children under 2 years of age and expressed in units/1,000 inhabitants <24 months old/year.

Incidence rates (IR) per 100,000 inhabitants of the corresponding age group were calculated for total IPD (children <15 years of age), total IPD stratified by age groups, IPD caused by PCV7 serotypes (PCV7-type IPD), IPD caused by PCV13 serotypes (PCV13-type IPD), and IPD caused by serotypes not included in PCV13 (non-PCV13-type IPD). Incidence rate ratios (IRRs), with their respective 95% confidence intervals, were calculated as ratios between IR in 2011–12 (last year of PCV13 in RIP)/IR in 2009–10 (last year of PCV7 in RIP), IR in 2013–14 (first year of the private period)/IR in 2012–13 (mixed public/private funding period), and IR in 2014–15 (last year of the private period)/IR in 2009–10 (last year of PCV7 in RIP). Comparisons were performed with the EPIDAT version 3.1.

The Triple Exponential Smoothing “Holt-Winters” (TES-HW) time-series model [23] was used to estimate IPD cases that would have occurred if changes in vaccination policies had not taken place.

## Results

Between May, 2012 and April, 2015, 113 IPD (31 empyema, 24 primary bacteremia, 21 bacteremic pneumonia, 16 meningitis, and 21 cases of other IPD) were included. When pooling these data with previously reported IPD cases [13–17] in all periods (May 2007 to April 2015) a total of 860 IPD were included in the study. Overall, empyema was the most frequent clinical presentation (294; 34.2%) followed by bacteremic pneumonia (235; 27.3%), primary bacteremia (121; 14.1%), meningitis (104; 12.1%) and others (106; 12.3%).

In a total of 196 (22.8%) cases, *S*. *pneumoniae* was identified by PCR (25 cases in the Private period); of them, 128 (65.3%) could be serotyped.

Estimated vaccine coverages in the different periods were: 95% (from 2007–8 to 2011–12), 82% (2012–13), 67% (2013–14) and 73% (2014–15).

**Fig 1:**
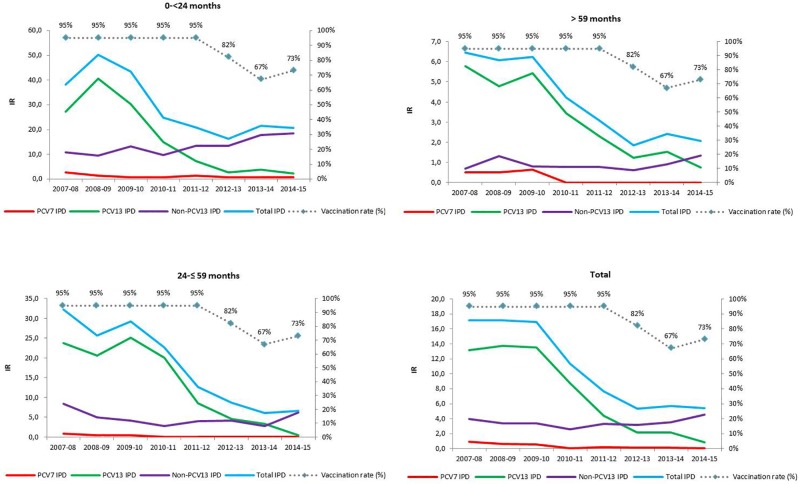
Incidence rates. Incidence rate (IR) of invasive pneumococcal disease in total (all children <15 years old) and in the different age groups. Percentages of vaccine coverage are shown as dotted lines. Data from 2007 to 2012 have been previously reported (references 13–17).

Fig 1 shows yearly IRs of PCV7-, PCV13-, non-PCV13-, and total IPDs for the different age groups. Table 1 shows globally and separately by age group, IRs for all study periods and IRRs. Overall, the IR of total IPD for all age groups decreased significantly, compared with 2009–10, by 55% in 2011–12, and 68% in 2014–15, due to a 68% and 93% reduction, respectively, in the IR of PCV13-type IPD, without significant changes in non-PCV13-type IPD. The highest reduction was observed in the group of children 24-≤59 months (77%).

**Table 1:** Per-age, no. (incidence rate) [95% confidence interval] and incidence rate ratio (IRR) [95% confidence interval] of all-type, PCV7-, PCV13- and non-PCV13- type IPD in children <15 years old in Madrid (impact population).

		PCV7 period^a^			PCV13 period^a^			Mixed^a^	Private period			
		2007–08	2008–09	2009–10	2010–11	2011–12	IRR 2011–12 / 2009–10	2012–13	2013–14	2014–15	IRR 2013–14 / 2012–13	IRR 2014–15 / 2009–10
% vaccine uptake		95%	95%	95%	95%	95%		82%	67%	73%		
**0-<24 months**	PCV7 IPD	4 (2.72) [0.09_5.36]	2 (1.36) [-0.51_3.22]	1 (0.66) [-0.63_1.95]	1 (0.66) [-0.63_1.94]	2 (1.34) [-0.51_3.2]	2.04 [0.11–120.24] p = 0.986	1 (0.71) [-0.67_2.09]	1 (0.75) [-0.71_2.2]	1 (0.77) [-0.73_2.26]	1.06 [0.01–82.96] p = 1.000	1.16 [0.02–91.25] p = 1.000
	PCV13 IPD	40 (27.24) [20.04_34.44]	60 (40.69) [32.76_48.62]	46 (30.35) [23.03_37.67]	23 (15.08) [9.4_20.76]	11 (7.4) [3.19_11.6]	0.24 [0.13–0.47] p<0.001	4 (2.83) [0.52_6.94]	5 (3.73) [-0.27_4.87]	3 (2.3) [0.52_6.94]	1.32 [0.28–6.66] p = 0.932	0.08 [0.02–0.24] p<0.001
	Non-PCV13 IPD	16 (10.9) [5.86_15.93]	14 (9.5) [4.76_14.23]	20 (13.2) [7.81_18.59]	15 (9.84) [5.11_14.56]	20 (13.45) [7.96_18.93]	1.02 [0.55–1.89] p = 0.922	19 (13.42) [7.8_19.03]	24 (17.92) [11.42_24.41]	24 (18.41) [11.76_25.06]	1.34 [0.73–2.44] p = 0.429	1.40 [0.77–2.53] p = 0.341
	Total IPD	56 (38.13) [30.28_45.99]	74 (50.19) [42.12_58.26]	66 (43.55) [35.66_51.45]	38 (24.92) [18.05_31.78]	31 (20.84) [14.32_27.37]	0.48 [0.31–0.73] p = 0.001	23 (16.24) [10.17_22.32]	29 (21.65) [14.67_28.62]	27 (20.71) [13.75_27.67]	1.33 [0.77–2.30] p = 0.372	0.48 [0.30–0.74] p = 0.001
**24-≤59 months**	PCV7 IPD	2 (0.93) [-0.35_2.22]	1 (0.46) [-0.44_1.35]	1 (0.46) [-0.44_1.35]	0 (0) [0_0]	0 (0) [0_0]	0.99 [0.01–77.81] p = 1.000	0 (0) [0_0]	0 (0) [0_0]	0 (0) [0_0]	1.02 [0.01–80.40] p = 1.000	1.04 [0.01–82.23] p = 1.000
	PCV13 IPD	51 (23.81) [18.11_29.52]	45 (20.61) [15.24_25.97]	55 (25.09) [19.35_30.83]	44 (20) [14.71_25.28]	19 (8.55) [4.88_12.23]	0.34 [0.20–0.58] p<0.001	10 (4.57) [1.8_7.34]	7 (3.28) [0.89_5.66]	1 (0.48) [-0.46_1.41]	0.72 [0.27–1.88] p = 0.665	0.02 [0.00–0.14] p<0.001
	Non-PCV13 IPD	18 (8.4) [4.69_12.12]	11 (5.04) [2.14_7.94]	9 (4.11) [1.48_6.73]	6 (2.73) [0.57_4.88]	9 (4.05) [1.46_6.64]	0.99 [0.39–2.50] p = 1.000	9 (4.11) [1.48_6.74]	6 (2.81) [0.59_5.02]	13 (6.21) [2.94_9.48]	0.68 [0.24–1.92] p = 0.641	1.51 [0.65–3.54] p = 0.454
	Total IPD	69 (32.22) [25.96_38.48]	56 (25.64) [19.85_31.44]	64 (29.2) [23.18_35.22]	50 (22.72) [17.19_28.26]	28 (12.61) [8.24_16.97]	0.43 [0.28–0.68] p<0.001	19 (8.68) [4.95_12.41]	13 (6.08) [2.88_9.29]	14 (6.69) [3.31_10.08]	0.70 [0.35–1.42] p = 0.414	0.23 [0.13–0.41] p<0.001
**>59 months**	PCV7 IPD	3 (0.51) [-0.07_1.08]	3 (0.49) [-0.06_1.05]	4 (0.64) [0.01_1.26]	0 (0) [0_0]	0 (0) [0_0]	0.23 [0.01–2.41] p = 0.346	0 (0) [0_0]	0 (0) [0_0]	0 (0) [0_0]	0.98 [0.01–76.84] p = 1.000	0.23 [0.01–2.34] p = 0.329
	PCV13 IPD	34 (5.77) [3.89_7.65]	29 (4.77) [3.08_6.46]	34 (5.44) [3.66_7.21]	22 (3.43) [2.02_4.84]	15 (2.29) [1.14_3.43]	0.42 [0.23–0.77] p = 0.006	8 (1.23) [0.38_2.08]	10 (1.51) [0.58_2.44]	5 (0.74) [0.09_1.39]	1.22 [0.48–3.10] p = 1.000	0.14 [0.05–0.35] p<0.001
	Non-PCV13 IPD	4 (0.68) [0.02_1.34]	8 (1.32) [0.41_2.22]	5 (0.8) [0.1_1.5]	5 (0.78) [0.1_1.46]	5 (0.76) [0.1_1.43]	0.95 [0.28–3.30] p = 1.000	4 (0.62) [0.01_1.22]	6 (0.91) [0.18_1.63]	9 (1.33) [0.47_2.2]	1.47 [0.35–7.08] p = 0.780	1.67 [0.56–4.98] p = 1.000
	Total IPD	38 (6.45) [4.47_8.43]	37 (6.08) [4.18_7.98]	39 (6.24) [4.34_8.13]	27 (4.21) [2.66_5.76]	20 (3.05) [1.73_4.37]	0.49 [0.29–0.84] p = 0.012	12 (1.85) [0.81_2.89]	16 (2.42) [1.25_3.59]	14 (2.07) [1_3.15]	1.31 [0.62–2.76] p = 0.610	0.33 [0.18–0.61] p<0.001
	PCV7 IPD	9 (0.95) [0.33_1.56]	6 (0.62) [0.12_1.11]	6 (0.6) [0.12_1.08]	1 (0.1) [-0.09_0.29]	2 (0.19) [-0.07_0.46]	0.32 [0.07–1.60] p = 0.270	1 (0.1) [-0.1_0.29]	1 (0.1) [-0.09_0.29]	1 (0.1) [-0.09_0.29]	1.00 [0.01–78.41] p = 1.000	0.16 [0.02–1.36] p = 0.119
	PCV13 IPD	125 (13.16) [11.01_15.3]	134(13.76) [11.6_15.92]	135(13.55) [11.43_15.68]	89 (8.78) [7.04_10.52]	45 (4.38) [3.13_5.64]	0.32 [0.23–0.45] p<0.001	22 (2.18) [1.28_3.08]	22 (2.18) [1.28_3.08]	9 (0.89) [0.31_1.46]	1.00 [0.55–1.80] p = 0.883	0.07 [0.03–0.13] p<0.001
**Total**	Non-PCV13 IPD	38 (4.0) [2.75_5.24]	33 (3.39) [2.25_4.52]	34 (3.41) [2.29_4.54]	26 (2.56) [1.59_3.54]	34 (3.31) [2.22_4.41]	0.97 [0.60–1.56] p = 0.998	32 (3.17) [2.09_4.25]	36 (3.57) [2.42_4.71]	46 (4.53) [3.25_5.81]	1.12 [0.70–1.81] p = 0.720	1.33 [0.85–2.07] p = 0.252
	Total IPD	163 (17.2) [14.76_19.55]	167 (17.1) [14.78_19.51]	169 (17.0) [14.63_19.29]	115 (11.3) [9.39_13.29]	79 (7.7) [6.07_9.33]	0.45 [0.35–0.59] p<0.001	54 (5.4) [3.97_6.74]	58 (5.7) [4.31_7.18]	55 (5.4) [4.03_6.81]	1.07 [0.74–1.55] p = 0.782	0.32 [0.24–0.43] p<0.001
**Population**	<24 months	146,856	147,443	151,547	152,498	148,721		141,587	133,965	130,373		
	24–≤59 months	214,176	218,377	219,204	220,052	222,127		218,833	213,659	209,240		
	>59 months	589,172	608,079	625,492	641,342	655,687		648,071	662,035	675,322		
	Total	950,204	973,899	996,243	1,013,892	1,026,535		1,008,491	1,009,659	1,014,935		

^a^Data previously published (references 13–17).

In children 0-<24 months, the IR of total IPD decreased significantly by 52% in 2011–12 due to a significant decrease (76%) in PCV13-type IPD without significant changes in non-PCV13-type IPD. However, when vaccination uptake fell to 67% in 2013–14, the IR non-significantly increased compared with 2012–13 (IRR: 1.33, 95%CI: 0.77–2.30; p = 0.372) due to non-significant increases in both PCV13-type (IRR: 1.32, 95%CI: 0.28–6.66; p = 0.932) and non-PCV13-type IPD (IRR: 1.34, 95%CI: 0.73–2.44; p = 0.429). A similar pattern was observed in children >59 months (total IPD IRR 2013–14 / 2012–13 of 1.31, 95%CI: 0.62–2.76; p = 0.610) (Table 1).

Table 2 shows by clinical presentation IRs and number of cases for all study periods and IRRs. IRs of empyema and bacteremic pneumonia decreased significantly both in 2011–12 (54% and 67%, respectively) and in 2014–15 (81% and 79%, respectively) with respect to 2009–10. IRs of meningitis decreased, but not significantly (46% in 2011–12 and 56% in 2014–15). IRs of primary bacteremia decreased non-significantly in 2011–12 (33% reduction) but in 2014–15 remained similar to 2009–10 with only two cases caused by PCV13 serotypes (serotypes 19A and 6C).

**Table 2:** Per-clinical presentation, no. (incidence rate) [95% confidence interval] and incidence rate ratio (IRR) [95% confidence interval] of IPD in children <15 years old in Madrid (impact population).

	PCV7 period^a^			PCV13 period^a^			Mixed^a^	Private period		
	2007–08	2008–09	2009–10	2010–11	2011–12	IRR 2011–12 / 2009–10	2012–13	2013–14	2014–15	IRR 2014–15 / 2009–10
% vaccine uptake	95%	95%	95%	95%	95%		82%	67%	73%	
**Meningitis**	23 (2.42) [1.44_3.4]	22 (2.26) [1.33_3.19]	18 (1.81) [0.98_2.63]	9 (0.89) [0.31_1.47]	10 (0.98) [0.37_1.57]	0.54 [0.25–1.17] p = 0.161	6 (0.59) [0.12_1.07]	8 (0.79) [0.25_1.34]	8 (0.79) [0.24_1.33]	0.44 [0.19–1.00] p = 0.070
**Empyema**	50 (5.26) [3.84_6.68]	50 (5.13) [3.75_6.52]	67 (6.73) [5.17_8.28]	42 (4.15) [2.92_5.37]	32 (3.12) [2.05_4.18]	0.46 [0.30–0.71] p<0.001	22(2.18) [1.28_3.08]	18 (1.78) [0.97_2.6]	13 (1.28) [0.59_1.97]	0.19 [0.11–0.35] p<0.001
**Bacteremic pneumonia**	62 (6.52) [4.95_8.1]	52 (5.34) [3.93_6.75]	47 (4.72) [3.4_6.03]	30 (2.96) [1.92_4]	16 (1.56) [0.8_2.32]	0.33 [0.19–0.58] p<0.001	7 (0.69) [0.18_1.21]	11 (1.09) [0.45_1.73]	10(0.99) [0.38_1.59]	0.21 [0.11–0.41] p<0.001
**Primary bacteremia**	19 (2.00) [1.11_2.89]	25 (2.57) [1.57_3.56]	16 (1.61) [0.83_2.39]	15 (1.48) [0.74_2.22]	11 (1.08) [0.44_1.7]	0.67 [0.31–1.44] p = 0.397	11 (1.09) [0.45_1.73]	8 (0.79) [0.25_1.34]	16 (1.58) [0.81_2.34]	0.98 [0.49–1.96] p = 0.901
**Other IPD**	9 (0.95) [0.33_1.56]	18 (1.85) [1_2.69]	21 (2.11) [1.22_3.00]	19 (1.88) [1.04_2.71]	10 (0.98) [0.37_1.57]	0.46 [0.22–0.98] p = 0.060	8 (0.79) [0.25_1.34]	13 (1.29) [0.59_1.98]	8 (0.79) [0.24_1.33]	0.37 [0.17–0.84] p = 0.023
**Population**	950,204	973,899	996,243	1,013,892	1,026,535		1,008,491	1,009,659	1,014,935	

^a^Data previously published (references 13–17).

Table 3 shows by age group serotype-specific IRs in 2009–10 and 2014–15, and IRRs 2014-15/2009-10 for the six added serotypes in PCV13. Overall, serotypes 1 and 19A decreased significantly both in 2011–12 (96% and 94%, respectively) (data not shown) and in 2014–15 (95% and 98%, respectively) with respect to 2009–10. The evolution of the six added serotypes in PCV13 along all study periods is shown per age group in S1 Table. In all age groups, these serotypes showed marked decreases over time, the decrease in serotype 19A (the most prevalent in the pre-PCV13 era in children <24 months) and in serotype 1 (the most prevalent in all other age groups in the pre-PCV13 era) being the most relevant. In the last study period, these six added serotypes caused only eight IPDs.

**Table 3:** Per-age, serotype-specific no. (incidence rate) [95% confidence interval] and incidence rate ratio (IRR) [95% confidence interval] of IPD in children <15 years old in Madrid (impact population).

Serotypes	0-<24 months		IRR 2014–15 / 2009–10	24-≤59 months		IRR 2014–15 / 2009–10	>59 months		IRR 2014–15 / 2009–10	Total		IRR 2014–15 / 2009–10
	2009–10^a^	2014–15		2009–10^a^	2014–15		2009–10^a^	2014–15		2009–10^a^	2014–15	
**1**	5 (3.3) [-0.2_6.8]	0 (0) [0_0]	0.23 [0.01–2.08) p = 0.297	25 (11.4) [5.2_17.6]	0 (0) [0_0]	0.04 [0.01–0.31] p<0.001	24 (3.84) [0.1_7.6]	3 (0.44) [-0.9_1.7]	0.12 [0.03–0.38] p<0.001	54 (5.42) [1_9.9]	3 (0.3) [-0.8_1.4]	0.05 [0.02–0.17] p<0.001
**19A**	27 (17.82) [10.3_25.3]	1 (0.77) [-0.9_2.5]	0.04 [0.01–0.32) p<0.001	17 (7.76) [2.5_13]	0 (0) [0_0]	0.06 [0.01–0.46] p<0.001	4 (0.64) [-0.9_2.2]	0 (0) [0_0]	0.23 [0.01–2.34] p = 0.329	48 (4.82) [0.6_9]	1 (0.1) [-0.5_0.7]	0.02 [0.00–0.15] p<0.001
**5**	1 (0.66) [-0.9_2.3]	0 (0) [0_0]	1.16 [0.02–91.25) p = 1.000	1 (0.46) [-0.9_1.8]	0 (0) [0_0]	1.05 [0.01–82.23] p = 1.000	2 (0.32) [-0.8_1.4]	0 (0) [0_0]	0.46 [0.01–8.90] p = 0.943	4 (0.4) [-0.8_1.6]	0 (0) [0_0]	0.25 [0.01–2.48] p = 0.364
**3**	6 (3.96) [0.1_7.8]	1 (0.77) [-0.9_2.5]	0.19 [0.02–1.61) p = 0.182	5 (2.28) [-0.6_5.2]	1 (0.48) [-0.9_1.8]	0.21 [0.00–1.87] p = 0.241	0 (0) [0_0]	2 (0.3) [-0.8_1.4]	1.85 [0.10–109.29] p = 1.000	11 (1.1) [-0.9_3.2]	4 (0.39) [-0.8_1.6]	0.36 [0.11–1.12] p = 0.110
**7F**	5 (3.3) [-0.2_6.8]	0 (0) [0_0]	0.23 [0.01–2.08) p = 0.297	6 (2.74) [-0.5_5.9]	0 (0) [0_0]	0.17 [0.02–1.45] p = 0.141	0 (0) [0_0]	0 (0) [0_0]	NA	11 (1.1) [-0.9_3.2]	0 (0) [0_0]	0.09 [0.01–0.69] p = 0.006
**6A**	1 (0.66) [-0,9_2,3]	0 (0) [0_0]	1.16 [0.02–91.25) p = 1.000	0 (0) [0_0]	0 (0) [0_0]	NA	0 (0) [0_0]	0 (0) [0_0]	NA	1 (0.1) [-0,5_0,7]	0 (0) [0_0]	0.98 [0.01–77.05] p = 1.000
**Population**	151,547	130,373		219,204	209,240		625,492	675,322		996,243	1,014,935	

^a^Data previously published (references 13–17).

Along the 8-year study period, the most common non-PCV13 serotypes causing IPD (each of them accounting for ≥5% of total non-PCV13-type IPD) were serotypes 15B (8.6%), 24F (6.8%), 23B (6.1%), 22F (5.7%), and 10A (5.0%). None of the non-PCV13 serotypes stood out for showing increases over time. In the last study period (2014–15), 19 different serotypes were identified among 46 non-PCV13 serotypes causing IPD.

**Fig 2:**
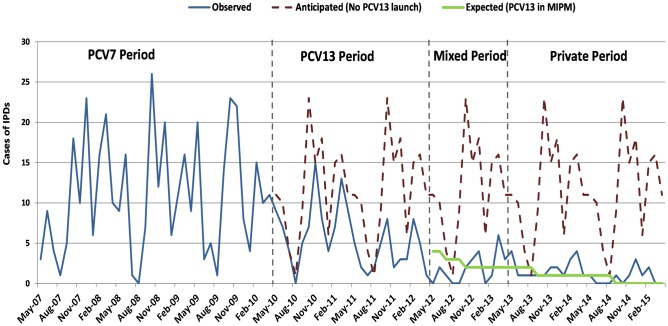
Evolution of cases of invasive pneumococcal disease (IPD) caused by serotypes included in the 13-valent pneumococcal conjugate vaccine (PCV13). Estimated evolution of PCV13-type IPD in relation to PCV13 introduction using the Holt-Winters exponential smoothing.

S2 Table shows the number of anticipated, observed and expected cases in the different study periods according to the forecasting model and Fig 2 shows the estimated evolution of PCV13 cases. In the absence of PCV13, IPDs would have been mostly caused by PCV13 serotypes. If PCV13 vaccination had continued in the RIP, according to the model, the decrease in cases of IPD observed after PCV13 introduction would have been even more pronounced than reported. In the private funding period up to 31 IPD (16 empyema, seven bacteremic pneumonia, two primary bacteremia, one meningitis and five other IPD) caused by PCV13 serotypes occurred: 11 by serotype 1, 14 by serotype 3, one by serotype 6B, three by serotype 7F, one by serotype 19A, and one by serotype 19F. According to the model, 20 cases by PCV13 serotypes would have occurred in that period if PCV13 had not been removed from RIP, thus, 11 out of 31 (35.5%) PCV13-type IPD could have been prevented. If this analysis of PCV13-type observed versus expected cases had been performed annually in the private funding period, 27.3% of cases (6 out of 22) would have been prevented in 2013–14 and 55.6% (5 out of 9) in 2014–15.

## Discussion

To the best of our knowledge, this is the first study that, by means of prospective active surveillance, analyses the effects of removing infant pneumococcal immunization with PCV13 from a fully-government funded program. During the two years of free infant universal pneumococcal vaccination with PCV13, infant vaccine uptakes reached 95% and there was a significant overall reduction of IPD (55%) in children younger than 15 years. During the 8-year study period (2007–15), all IPD were significantly reduced (68% reduction) through a near elimination of PCV13-type IPD (93% reduction), particularly IPD by serotypes 1 and 19A which, before the introduction of PCV13, accounted for 60.4% of all IPD (Table 1 and S1 Table). The decrease in IPD occurred in vaccinated and unvaccinated children suggesting that PCV13 induces herd immunity. However, vaccine unrelated secular trends [24] could also have influenced the decrease of some serotypes, as has also been suggested by other authors for serotypes 1 and 5, both among children [1] and adults [25].

After the exclusion of PVC13 from the fully government-funded vaccination program, vaccine uptake dropped to 67%. Although IR of all IPD and of PCV13-type IPD continued to decrease in children 24-≤59 months (children vaccinated with PCV13 during the free vaccination program), in children <2 years of age (the target group for vaccination) and children >5 years (children unimmunized with PCV13) the decline in PCV13-type IPD stagnated.

Nasopharyngeal colonization, which begins in the first months of age and peaks at 3 years, plays a central role in the epidemiology of pneumococcal infections [26]. Reduction of nasopharyngeal colonization by PCVs decreases transmission of vaccine types, thereby amplifying the protective effect of the vaccine (herd immunity) since it has been hypothesized that pneumococcal serotypes with greater capacity to cause disease (PCV13 serotypes) are replaced by less pathogenic serotypes [12]. It has been estimated that indirect protection induced by PCV in unimmunized people prevents twice as many cases as direct protection in immunized individuals [27].

Significant reduction of nasopharyngeal colonization by vaccine types in unimmunized people appears when vaccine coverage in children reaches 65–70% [28]. In the Region of Madrid, vaccine coverage fell to 67% and it could be hypothesized that the reverse phenomenon of loss of herd immunity could have commenced in children older than 5 years who were not immunized.

Our data suggest that the drop in vaccine coverage below the threshold from which herd immunity can be expected could lead to a rapid increase in IPD incidence in unimmunized people. The effect of herd immunity on IPD is seen within just a few months after PCV13 introduction [29]. In our study, the incidence of IPD by vaccine types fell from 5.44/100,000 to 3.43/100,000 in unimmunized children >59 months of age, only one year after introduction of PCV13. These data suggest that herd immunity may quickly appear as long as high vaccine coverage is maintained [30,31], although confounding factors as serotypes secular trends cannot be excluded. Nevertheless, as has been suggested [1,25], PCVs may intensify and support natural secular trends.

It has been reported that the overall reduction of IPD prevented by PCVs is only partly offset by serotype replacement [1,32]. Our data confirm this; the drastic reduction in the IR of PCV13-type IPD was accompanied by a non-significant increase in non-PCV13-type IPD. Unlike the rapid increase in non-PCV7 serotypes after PCV7 introduction, particularly of serotype 19A [13–17], in our study none of the non-PCV13 serotypes stood out above the rest. The IR of non-PCV13-type IPD was higher in infants younger than 24 months of age, likely reflecting more rapid and pronounced changes in nasopharyngeal carriage in vaccinated people. Serotype replacement after PCV13 implementation is linked to non-PCV13 serotypes [32,33] colonizing vacant nasopharyngeal niches previously occupied by PCV13 serotypes. Non-PCV13 serotypes have replaced PCV13 serotypes in the nasopharynx [10,34], and the emergence of non-PCV13 serotypes causing IPD has been found in England and Wales in children younger than 5 years, four years after the introduction of PCV13 [4], but not in the USA [2]. The effect of nasopharyngeal changes on the IR of IPD depends on the invasive potential of emergent serotypes. Non-PCV13 serotypes are less invasive than PCV13 serotypes [31]; one may hypothesize that this is related to the slight increase in primary bacteremia (but not other clinical forms) caused by non-PCV13 serotypes in the present study. Primary bacteremia is a clinical form of pneumococcal infection highly related to physiological incapacity to respond to polysaccharide antigens leading to high susceptibility to pneumococcal infection, as occurs in children <2 years of age.

Epidemiologically, according to our forecast data, the vaccination impact would have been greater in the period 2014–2015 with >50% IR reduction (versus observed) in PCV13-type IPD, without changes in non-PCV13-types. Clinically, avoidable IPD would have been mostly empyema and bacteremic pneumonia, probably because of the different tropism of pneumococcal serotypes, with serotype 1 linked to respiratory presentations.

One study limitation is the estimation of vaccine coverages considering a 2+1 dose schedule instead of the recommended 3+1, which may falsely increase vaccination coverages. However, it seemed appropriate since adherence was probably suboptimal in the private period, as previously reported by other authors in Spain and other European countries [8]. Another limitation is specific imperfect matches between vaccination and study periods. In the strict sense, the private vaccination period ran from May, 2013 to January, 2015, while the last study period ended in April, 2015. Nevertheless, epidemiologically, these discrepancies have no substantial weight since all IPDs in hospitalized children in Madrid (>6 million inhabitants) were included. Finally, only isolates belonging to serotypes 1, 3, 4, 5, 6, 7F, 14, 19A, and 19F could be serotyped by the real-time PCR assay used for serotyping of pneumococci identified by PCR; therefore, we cannot dismiss the possibility that the IPDs diagnosed by PCR were caused by pneumococci belonging to some PCV13 serotypes as 4, 9V, 18C, and 23F. However, since only 68 isolates identified by PCR could not be serotyped (representing only 7.9% of total IPD cases), this possibility is of limited importance.

## Conclusions

The present study strongly suggests the efficacy of a fully government-funded program for universal vaccination against pneumococci, which guarantees high vaccine uptake levels, and consequently a great impact on IPD through both direct and indirect vaccination effects. Lack of completion of vaccination schedules increases when the vaccine is not included in free public immunization programs, making costs an issue for parents [8], disinclining them to pay for booster doses which are sometimes wrongly perceived as “less important”. The fall in vaccine coverage below the threshold necessary to maintain herd immunity is rapidly followed by a loss of indirect protection effect in unimmunized children.

Although secular natural trends of serotypes have a potential role that cannot be ignored, the results of our forecast data show that the maintenance of PCV13 in RIP would have reduced the incidence of PCV13-type IPD to a greater degree without significant increased incidences in non-PCV13-type IPD. Therefore, the reintroduction of PCV13 in the RIP in May, 2015 was an appropriate measure, the impact of which warrants future monitoring.

## Supporting information

S1 Table Per-age evolution of the six added serotypes in PCV13. (DOCX)

S2 Table No. cases of PCV13- and non-PCV13- type IPD in the different study periods: Anticipated (no PCV13 launch) vs. Observed vs. Expected (PCV13 included in RIP). (DOCX)
