# Supplementary material for: Effect of the different 13-valent pneumococcal conjugate vaccination uptakes on the invasive pneumococcal disease in children: Analysis of a hospital-based and population-based surveillance study in Madrid, Spain, 2007-2015
Source: PLoS One. 2017 Feb 16;12(2):e0172222. doi: 10.1371/journal.pone.0172222 (PMC5312951; doi:10.1371/journal.pone.0172222)
Supplement: S1 Table — (DOCX) [file pone.0172222.s001.docx]

**Table S1**. **Per-age evolution of the six added serotypes in PCV13**

|  | **Serotypes** | **PCV7 period ^a^** | | | | | | **PCV13 period ^a^** | | | | **Mixed ^a^** | | **Private period** | | | |
| --- | --- | --- | --- | --- | --- | --- | --- | --- | --- | --- | --- | --- | --- | --- | --- | --- | --- |
|  |  | **2007-08** | | **2008-09** | | **2009-10** | | **2010-11** | | **2011-12** | | **2012-13** | | **2013-14** | | **2014-15** | |
|  |  | **n (IR)** | **IC95%** | **n (IR)** | **IC95%** | **n (IR)** | **IC95%** | **n (IR)** | **IC95%** | **n (IR)** | **IC95%** | **n (IR)** | **IC95%** | **n (IR)** | **IC95%** | **n (IR)** | **IC95%** |
| **0-<24 months** | **1** | 4 (2.72) | [0.1_5.4] | 6 (4.07) | [0.9_7.3] | 5 (3.3) | [0.5_6.1] | 0 (0.0) | [0_0] | 2 (1.34) | [-0.5_3.2] | 0 (0.0) | [0_0] | 1 (0.75) | [-0.7_2.2] | 0 (0.0) | [0_0] |
|  | **19A** | 13 (8.85) | [4.3_13.5] | 34 (23.06) | [16.3_29.9] | 27 (17.82) | [11.7_23.9] | 16 (10.49) | [5.6_15.4] | 4 (2.69) | [0.1_5.3] | 2 (1.41) | [-0.5_3.4] | 0 (0.0) | [0_0] | 1 (0.77) | [-0.7_0] |
|  | **5** | 9 (6.13) | [2.3_10] | 6 (4.07) | [0.9_7.3] | 1 (0.66) | [-0.6_2] | 1 (0.66) | [-0.6_1.9] | 0 (0.0) | [0_0] | 0 (0.0) | [0_0] | 0 (0.0) | [0_0] | 0 (0.0) | [0_0] |
|  | **3** | 0 (0.0) | [0_0] | 4 (2.71) | [0.1_5.3] | 6 (3.96) | [0.9_7.1] | 3 (1.97) | [-0.2_4.2] | 2 (1.34) | [-0.5_3.2] | 0 (0.0) | [0_0] | 3 (2.24) | [-0.3_4.7] | 1 (0.77) | [-0.7_0] |
|  | **7F** | 7 (4.77) | [1.3_8.2] | 8 (5.43) | [1.8_9.1] | 5 (3.3) | [0.5_6.1] | 1 (0.66) | [-0.6_1.9] | 1 (0.67) | [-0.6_2] | 1 (0.71) | [-0.7_2.1] | 0 (0.0) | [0_0] | 0 (0.0) | [0_0] |
|  | **6A** | 3 (2.04) | [-0.3_4.3] | 0 (0.0) | [0_0] | 1 (0.66) | [-0.6_2] | 1 (0.66) | [-0.6_1.9] | 0 (0.0) | [0_0] | 0 (0.0) | [0_0] | 0 (0.0) | [0_0] | 0 (0.0) | [0_0] |
| **24-≤59 months** | **1** | 18 (8.4) | [4.7_12.1] | 28 (12.82) | [8.4_17.3] | 25 (11.4) | [7.2_15.6] | 20 (9.09) | [5.3_12.9] | 9 (4.05) | [1.5_6.6] | 2 (0.91) | [-0.3_2.2] | 1 (0.47) | [-0.5_1.4] | 0 (0.0) | [0_0] |
|  | **19A** | 6 (2.8) | [0.6_5] | 3 (1.37) | [-0.2_2.9] | 17 (7.76) | [4.2_11.3] | 12 (5.45) | [2.5_8.5] | 1 (0.45) | [-0.4_1.3] | 0 (0.0) | [0_0] | 0 (0.0) | [0_0] | 0 (0.0) | [0_0] |
|  | **5** | 16 (7.47) | [4_11] | 7 (3.21) | [0.9_5.5] | 1 (0.46) | [-0.4_1.4] | 2 (0.91) | [-0.4_2.2] | 0 (0.0) | [0_0] | 0 (0.0) | [0_0] | 0 (0.0) | [0_0] | 0 (0.0) | [0_0] |
|  | **3** | 4 (1.87) | [0.1_3.7] | 3 (1.37) | [-0.2_2.9] | 5 (2.28) | [0.3_4.3] | 5 (2.27) | [0.3_4.2] | 4 (1.8) | [0.1_3.6] | 7 (3.2) | [0.9_5.5] | 6 (2.81) | [0.6_5] | 1 (0.48) | [-0.5_0] |
|  | **7F** | 5 (2.33) | [0.3_4.4] | 3 (1.37) | [-0.2_2.9] | 6 (2.74) | [0.6_4.9] | 5 (2.27) | [0.3_4.2] | 5 (2.25) | [0.3_4.2] | 1 (0.46) | [-0.4_1.4] | 0 (0.0) | [0_0] | 0 (0.0) | [0_0] |
|  | **6A** | 0 (0.0) | [0_0] | 0 (0.0) | [0_0] | 0 (0.0) | [0_0] | 0 (0.0) | [0_0] | 0 (0.0) | [0_0] | 0 (0.0) | [0_0] | 0 (0.0) | [0_0] | 0 (0.0) | [0_0] |
| **> 59 months** | **1** | 14 (2.38) | [1.2_3.6] | 16 (2.63) | [1.4_3.9] | 24 (3.84) | [2.3_5.3] | 17 (2.65) | [1.4_3.9] | 15 (2.29) | [1.1_3.4] | 7 (1.08) | [0.3_1.9] | 6 (0.91) | [0.2_1.6] | 3 (0.44) | [-0.1_0.4] |
|  | **19A** | 4 (0.68) | [0_1.3] | 2 (0.33) | [-0.1_0.8] | 4 (0.64) | [0_1.3] | 0 (0.0) | [0_0] | 0 (0.0) | [0_0] | 0 (0.0) | [0_0] | 0 (0.0) | [0_0] | 0 (0.0) | [0_0] |
|  | **5** | 9 (1.53) | [0.5_2.5] | 4 (0.66) | [0_1.3] | 2 (0.32) | [-0.1_0.8] | 3 (0.47) | [-0.1_1] | 0 (0.0) | [0_0] | 0 (0.0) | [0_0] | 0 (0.0) | [0_0] | 0 (0.0) | [0_0] |
|  | **3** | 1 (0.17) | [-0.2_0.5] | 1 (0.16) | [-0.2_0.5] | 0 (0.0) | [0_0] | 0 (0.0) | [0_0] | 0 (0.0) | [0_0] | 1 (0.15) | [-0.1_0.5] | 1 (0.15) | [-0.1_0.5] | 2 (0.3) | [-0.1_0.2] |
|  | **7F** | 2 (0.34) | [-0.1_0.8] | 3 (0.49) | [-0.1_1.1] | 0 (0.0) | [0_0] | 1 (0.16) | [-0.2_0.5] | 0 (0.0) | [0_0] | 0 (0.0) | [0_0] | 3 (0.45) | [-0.1_1] | 0 (0.0) | [0_0] |
|  | **6A** | 1 (0.17) | [-0.2_0.5] | 0 (0.0) | [0_0] | 0 (0.0) | [0_0] | 1 (0.16) | [-0.2_0.5] | 0 (0.0) | [0_0] | 0 (0.0) | [0_0] | 0 (0.0) | [0_0] | 0 (0.0) | [0_0] |
| **Total** | **1** | 36 (3.79) | [2.6_5] | 50 (5.13) | [3.8_6.5] | 54 (5.42) | [4_6.8] | 37 (3.65) | [2.5_4.8] | 26 (2.53) | [1.6_3.5] | 9 (0.89) | [0.3_1.5] | 8 (0.79) | [0.3_1.3] | 3 (0.3) | [0_0.3] |
|  | **19A** | 23 (2.42) | [1.4_3.4] | 39 (4) | [2.8_5.2] | 48 (4.82) | [3.5_6.2] | 28 (2.76) | [1.8_3.8] | 5 (0.49) | [0.1_0.9] | 2 (0.2) | [-0.1_0.5] | 0 (0.0) | [0_0] | 1 (0.1) | [-0.1_0] |
|  | **5** | 34 (3.58) | [2.4_4.8] | 17 (1.75) | [0.9_2.6] | 4 (0.4) | [0_0.8] | 6 (0.59) | [0.1_1.1] | 0 (0.0) | [0_0] | 0 (0.0) | [0_0] | 0 (0.0) | [0_0] | 0 (0.0) | [0_0] |
|  | **3** | 5 (0.53) | [0.1_1] | 8 (0.82) | [0.3_1.4] | 11 (1.1) | [0.5_1.8] | 8 (0.79) | [0.2_1.3] | 6 (0.58) | [0.1_1.1] | 8 (0.79) | [0.2_1.3] | 10 (0.99) | [0.4_1.6] | 4 (0.39) | [0_0.4] |
|  | **7F** | 14 (1.47) | [0.7_2.2] | 14 (1.44) | [0.7_2.2] | 11 (1.1) | [0.5_1.8] | 7 (0.69) | [0.2_1.2] | 6 (0.58) | [0.1_1.1] | 2 (0.2) | [-0.1_0.5] | 3 (0.3) | [0_0.6] | 0 (0.0) | [0_0] |
|  | **6A** | 4 (0.42) | [0_0.8] | 0 (0.0) | [0_0] | 1 (0.1) | [-0.1_0.3] | 2 (0.2) | [-0.1_0.5] | 0 (0.0) | [0_0] | 0 (0.0) | [0_0] | 0 (0.0) | [0_0] | 0 (0.0) | [0_0] |

^a^Data previously published (references 13-17)
