# Supplementary material for: Effect of the different 13-valent pneumococcal conjugate vaccination uptakes on the invasive pneumococcal disease in children: Analysis of a hospital-based and population-based surveillance study in Madrid, Spain, 2007-2015
Source: PLoS One. 2017 Feb 16;12(2):e0172222. doi: 10.1371/journal.pone.0172222 (PMC5312951; doi:10.1371/journal.pone.0172222)
Supplement: S2 Table — (DOCX) [file pone.0172222.s002.docx]

**Table S2**. **No. cases of PCV13- and non-PCV13- type IPD in the different study periods: Anticipated (no PCV13 launch) vs. Observed vs. Expected (PCV13 included in RIP)**

|  | **PCV7 period** | | | **PCV13 period** | | **Mixed** | **Private period** | |
| --- | --- | --- | --- | --- | --- | --- | --- | --- |
|  | **2007-08** | **2008-09** | **2009-10** | **2010-11** | **2011-12** | **2012-13** | **2013-14** | **2014-15** |
| **Anticipated** |  |  |  | 175 [171_179] | 175 [171_179] | 175 [171_179] | 175 [171_179] | 175 [171_179] |
| **PCV13-type** |  |  |  | 139 [135_143] | 139 [135_143] | 139 [135_143] | 139 [135_143] | 139 [135_143] |
| **Non-PCV13-type** |  |  |  | 43 [42_44] | 43 [42_44] | 43 [42_44] | 43 [42_44] | 43 [42_44] |
| **Observed** | 164 [159_169] | 166 [160_172] | 170 [164_176] | 114 [111_117] | 79 [77_81] | 54 [52_56] | 59 [58_60] | 54 [52_56] |
| **PCV13-type** | 126 [121_131] | 133 [128_138] | 136 [131_141] | 88 [85_91] | 45 [43_47] | 22 [21_23] | 22 [21_23] | 9 [8_10] |
| **Non-PCV13-type** | 38 [37_39] | 33 [32_34] | 34 [33_35] | 26 [25_27] | 34 [33_35] | 32 [31_33] | 37 [36_38] | 45 [43_47] |
| **Expected** |  |  |  |  |  | 64 [62_66] | 58 [56_60] | 50 [43_57] |
| **PCV13-type** |  |  |  |  |  | 31 [30_32] | 16 [16_16] | 4 [3_5] |
| **Non-PCV13-type** |  |  |  |  |  | 42 [41_43] | 42 [41_43] | 42 [39_45] |
